# Antigenic mimicry of mammalian oligomannose by a naturally occurring bacterial oligosaccharide and its implications for HIV vaccine design

**DOI:** 10.1186/1742-4690-9-S2-P337

**Published:** 2012-09-13

**Authors:** C De Castro, BE Clark, K Auyeung, A Marzaioli, RL Stanfield, IA Wilson, R Pantophlet

**Affiliations:** 1 University of Napoli Federico II, Napoli, Italy; 2 The Scripps Research Institute, La Jolla, CA, USA; 3 Simon Fraser University, Burnaby, Canada

## Background

Oligomannose sugars on gp120 are of significance to HIV vaccine design because of the description of the broadly neutralizing mAb 2G12 and, more recently, of the potent and broadly neutralizing ‘PGT’ series of mAbs, which bind with high affinity to clusters of oligomannose moieties on gp120. Attempts to elicit oligomannose-specific antibodies have focused mainly on immunizing with antigenic clusters of synthetic oligomannose or natural oligomannose. These strategies have had limited success, suggesting that alternative approaches are needed. Here, we present the surface lipo-oligosaccharide (LOS) of Rhizobium radiobacter Rv3 that antigenically mimics the 2G12 epitope, as one potential new avenue of exploration.

## Methods

The chemical structure of the Rv3 OS was determined by NMR and its antigenic similarity to the 2G12 epitope determined by ELISA and X-ray crystallography. Immunogenicity of the Rv3 OS and its ability to elicit antibodies of 2G12-like specificity was assessed by immunizing mice with heat-killed Rv3 bacteria.

## Results

The detailed chemical analysis of the Rv3 OS revealed that its carbohydrate backbone consists of a unique tetramannose backbone that is analogous to the D1 arm of mammalian oligomannose. 2G12 bound with at least similar affinity to purified Rv3 OS as reported for oligomannose. The 2.4 Å-structure of the 2G12:Rv3 OS complex shows that 2G12 contacts all four mannosyl moieties in the Rv3 backbone that mimic the D1 arm, with the majority of contacts occurring with the terminal mannose disaccharide. Antibodies elicited by immunizing with heat-killed bacteria bound a synthetic tetramannose epitope and monomeric gp120.

## Conclusion

Although the elicited antibodies failed to exhibit the desired neutralizing activity, our data suggest that presentation of an antigenic analog of mammalian oligomannose in a bacterial context presents a novel avenue for pursuing immunogens to elicit oligomannose-specific HIV neutralizing antibodies. The development of Rv3-based bioconjugates using human carbohydrate vaccine carriers is ongoing.

